# Ambient temperature and ozone exposure increases the risk of preterm birth in Northeast China: a time series analysis

**DOI:** 10.3389/fpubh.2025.1712825

**Published:** 2025-12-05

**Authors:** Wanyan Geng, Jing Yuan, Jiancheng Li, Xinyue Wu, Hongxin Xu, Yuhui Sun, Haiyu Zhang, Tao Song, Yan Zhao

**Affiliations:** 1 Department of Nutrition and Food Hygiene, The National Key Discipline, School of Public Health, Harbin Medical University, Harbin, China; 2 Department of Obstetrics, The First Affiliated Hospital of Harbin Medical University, Harbin, China; 3 Department of Cardiology, The First Affiliated Hospital of Harbin Medical University, Harbin, China; 4 Department of Gynecology, The First Affiliated Hospital of Harbin Medical University, Harbin, China

**Keywords:** preterm birth, ambient temperature, humidex, ozone, distributed lag non-linear model

## Abstract

**Background:**

Prenatal exposure to meteorological factors and air pollutants may increase preterm birth (PTB) risk, but existing research is limited and inconsistent. Heilongjiang Province is located in Northeast China and is the northernmost and highest latitude province. Yet the association between meteorological factors, air pollutants, and PTB in this region remains unclear.

**Methods:**

PTB cases were collected between July 1, 2019, and June 30, 2023. Meteorological factors were collected for the same period. The exposure-response relationships and lagged effects of meteorological factors and air pollutants on PTB were analyzed using the Distributed Lag Non-linear Model (DLNM), with a maximum lag of 30 days.

**Results:**

A total of 3,267 PTB cases were included. Low and extremely low ambient temperatures were associated with an increased risk of PTB at lag day 20 (RR = 1.09, 95% CI: 1.01–1.18 and RR = 1.09, 95% CI: 1.02–1.16, respectively). In contrast, extremely high ambient temperatures were associated with an increased risk of PTB at lag day 0 (RR = 1.12, 95% CI: 1.00–1.25). An extremely high Humidex was associated with an increased risk of PTB at lag day 0 (RR = 1.15, 95% CI: 1.01–1.30). For ozone, each IQR increase (27.8 μg/m^3^) was associated with an increased risk of PTB, with the highest risk observed at lag day 0 (RR = 1.08, 95% CI: 1.01–1.15).

**Conclusion:**

This study indicated that extreme temperatures, as well as an extremely high Humidex and increased ozone concentrations, may be associated with an increased risk of PTB, with significant lag effects.

## 1 Introduction

Preterm birth (PTB) is defined as the delivery of a live infant before 37 completed weeks of gestation. Globally, PTB is one of the leading causes of neonatal morbidity and mortality. Over recent decades, its incidence has risen, driven by changes in lifestyle, environmental exposures, and other factors. PTB poses a significant threat to neonatal health, frequently resulting in complications affecting the respiratory, digestive, and nervous systems, as well as impairments in growth and development (1). In 2020, the number of preterm births in China ranked fourth in the world, with an annual PTB rate of around 6.1% between 2010 and 2020. In 2019, approximately 900,000 children worldwide died from complications of PTB, accounting for 16.6% of the mortality rate for children under 5 years old (2). An estimated 13.4 million babies were born too early in 2020, accounting for 9.9% of all births (3). Consequently, PTB has emerged as a major public health challenge, not only in China but worldwide. Pregnancy is a unique physiological period characterized by profound physical and psychological changes that influence both maternal and fetal health. Although certain risk factors, such as a history of PTB, multiple pregnancies, pregnancy complications, and infections, have been well confirmed, the etiology of most PTB cases remains unclear (4). In recent years, the impact of exposure to environmental risk factors during pregnancy on PTB has attracted widespread attention. Notably, pregnant individuals and fetuses are more susceptible to the adverse effects of extreme temperatures and air pollution exposure than the general population (5).

The etiology of PTB is intricate and exhibits regional variability, with current research findings often showing inconsistencies. Studies have shown that maternal exposure to extreme temperatures during pregnancy may increase the risk of PTB (6). Specifically, one study reported that exposure to high ambient temperatures during pregnancy was associated with a higher risk of PTB (7). Conversely, another study suggested that high temperatures may be a protective factor for PTB, whereas low temperatures may be a risk factor (8). Additionally, a large-scale population study in northern China identified that high Humidex (HMI) was associated with PTB (9). In Beijing, China, research has demonstrated that both short-term and long-term exposure to PM_2.5_ is associated with PTB. The PM_2.5_ concentration in Beijing is relatively high, and its impact varies seasonally (10). An observational birth cohort study in Detroit, Michigan, USA, found a higher correlation between PM_10_, but not NO_2_ or PM_2.5_, and PTB (11). A time series study in Tehran, Iran, showed that for each 0.8-unit increase in PM2.5 and NO2, the risk of PTB increased by 0.8 and 0.6%, respectively (12). In rural Henan Province, China, research demonstrated a positive correlation between PTB risk and exposure to PM_10_, PM_2.5_, SO_2_, NO_2_, and CO, but a negative correlation with ozone exposure (13). Conversely, a study in Chongqing suggested that short-term exposure to low concentrations of CO might have a protective effect on PTB, and prolonged low-level exposure could lower the risk of both preterm and extremely PTB (14). Several studies have shown that high levels of ozone exposure during pregnancy increase the risk of PTB (15–17). However, this association is not consistently observed, as other studies have failed to detect a significant correlation between ozone levels and PTB (18).

The above studies highlight that findings on the association between PTB and exposure to temperature extremes and air pollutants remain inconsistent. To address this gap, this study explores the relationships between PTB, meteorological factors, and air pollutants in cold regions, using Heilongjiang Province as a representative case. A time-series analysis of PTB data from Heilongjiang is employed to evaluate the associations between meteorological and air pollution exposures and PTB risk.

## 2 Materials and methods

### 2.1 Description of study populations

Heilongjiang Province is located in Northeast China and is the northernmost and highest latitude province with a unique geographical location and natural features, and belongs to the cold temperate and temperate continental monsoon climate, with average minimum winter temperatures reaching −30 °C. The daily number of preterm births was obtained from July 1, 2019, to June 30, 2023, from the First Affiliated Hospital of Harbin Medical University (a large comprehensive medical center) and the Harbin Children’s Hospital (a large specialized pediatric hospital) in Heilongjiang Province, representing a subset of PTB cases in the region. Both hospitals are situated in Harbin, the provincial capital of Heilongjiang. Demographic variables included maternal age and home address, as well as the newborn’s sex, gestational weeks, date of birth, and birth weight. WHO defines preterm as babies born alive before 37 weeks of pregnancy are completed. Gestational weeks were determined based on maternal self-reported last menstrual period (LMP) and first-trimester ultrasound examination. We excluded pregnant women with a home address outside of Heilongjiang Province, stillbirths, twin pregnancies, and multiple pregnancies. The final study population consisted of 3,267 women with singleton live births (Supplementary Figure S1). This study was approved by the Ethics Committee of the First Affiliated Hospital of Harbin Medical University (2025358). All data were anonymized and contained no personally identifiable information.

### 2.2 Meteorological data

We obtained local meteorological and air pollutant data from the China Integrated Information Service System (CIMISS) provided by the China Meteorological Data Service Center. There were 54 air quality monitoring stations within a 200-kilometer radius of Harbin. However, no stations were located in the Great Khingan Mountains. To ensure data consistency and accuracy, cases from this region were excluded from the analysis. Because the daily mean temperature better reflects the health status of the human body than the daily maximum or minimum temperatures, environmental temperature exposure was defined as the daily mean temperature (Temp) (19). Relative humidity exposure was defined as the daily mean relative humidity exposure (RH). For ozone (O_3_) exposure, we used the maximum daily 8-h average concentration. The daily mean concentrations of particulate matter <2.5 μm (PM_2.5_), particulate matter <10 μm (PM_10_), nitrogen dioxide (NO_2_), sulfur dioxide (SO_2_), and carbon monoxide (CO) were used as the exposure to air pollutants. Missing data were replaced using the nearest available station. Humidex (HMI) was defined as a comprehensive indicator reflecting the combined effects of temperature and humidity. When HMI exceeds 30, the human body will feel discomfort (20). Meteorological exposures were categorized as follows: extremely low (<5th percentile), extremely high (>95th percentile), low (<25th percentile), and high (>75th percentile). For air pollutants, we examined the risks of PTB associated with an interquartile range (IQR) increase in different lag days. The calculation formula for HMI used in this study was obtained from CSGNetwork,^1^ as follows:


Humidex=Temp+(5/9)∗(6.11∗10^((7.5∗Temp)/(237+Temp))∗(RH/100)−10)

Where Temp is the daily average temperature (°C), and RH is the daily relative humidity (%).

In this study, we first conducted descriptive statistical analysis on the characteristics of the cases and meteorological factors. Considering the potential nonlinear and lagged effects of temperature and air pollutants on PTB, we employed a quasi-Poisson generalized additive model (GAM) incorporating a distributed lag nonlinear model (DLNM) to estimate the exposure lag response relationships between temperature and air pollutants exposure and PTB (21, 22). GAM has been widely used to analyze the impact of air pollution on human health; the daily number of PTB events was a low-probability event and followed a Poisson distribution. The DLNM, based on the framework of GAM, introduced the core idea of cross-basis functions, which can analyze complex exposure lag response relationships. Based on prior literature, we adopted a 30-day lag period to fully capture the delayed effects of temperature and air pollutants on PTB. The model’s degrees of freedom were determined using the minimum Akaike Information Criterion (AIC). The df range we tested spanned from 2 to 8. Natural cubic spline curves were used to control for seasonal and long-term trends, and the day of the week (DOW) and public holiday indicators were incorporated as covariates. The model used in this study is expressed as follows:


$$\begin{array}{l} \log[E(\mathrm{Yt})]=\alpha +\mathrm{\beta Tt},l+\mathrm{ns}\;(\text{time},7^{*}\;\text{year})\\ +\mathrm{ns}\;(\mathrm{RH},3)+\gamma \text{Dowt}+\mu \text{Holidayt} \end{array}$$

Where Yt represents the daily number of preterm births on day t, α is the intercept, β is the regression coefficient, Tt,l are matrices representing temperature, air pollutants, and lag days, and RH is the relative humidity. ns (time, 7 * year) were used to regulate long-term trends and seasonal variations, DOW and Holiday were used to adjust for week effects and holiday impacts. The reference value was the median. Using P50 as a reference benchmark, we studied the relative risk (RR) and its 95% confidence interval (95% CI) associated with the single-day lag and cumulative lagged effects of meteorological factors on PTB. For air pollutants, we used P25 as the reference to study their RR and 95% CI for single-day and cumulative lag effects on PTB. In addition, stratified analyses were conducted by maternal age, gestational weeks, and season. In Heilongjiang Province, the heating season lasted approximately 6 months (October 20 to April 20 of the following year). The season was divided into warm (April 21 to October 19) and cold (October 20 to April 20) in this study. The Pearson correlation coefficient was applied to explore the correlation between meteorological factors and air pollutants, with a coefficient ≥0.7 considered a strong correlation. Sensitivity analyses were conducted by performed by constructing a dual pollutant model and changing the degrees of freedom of the model. A correlation coefficient ≥0.7 between meteorological factors and air pollutants was not included in the dual pollutant model to avoid multicollinearity.

All statistical tests were two-sided tests, with *p* < 0.05 considered statistically significant. All analyses were conducted through the “dlnm” package in R software (version 4.3.2).

## 3 Results

**Figure 1:**
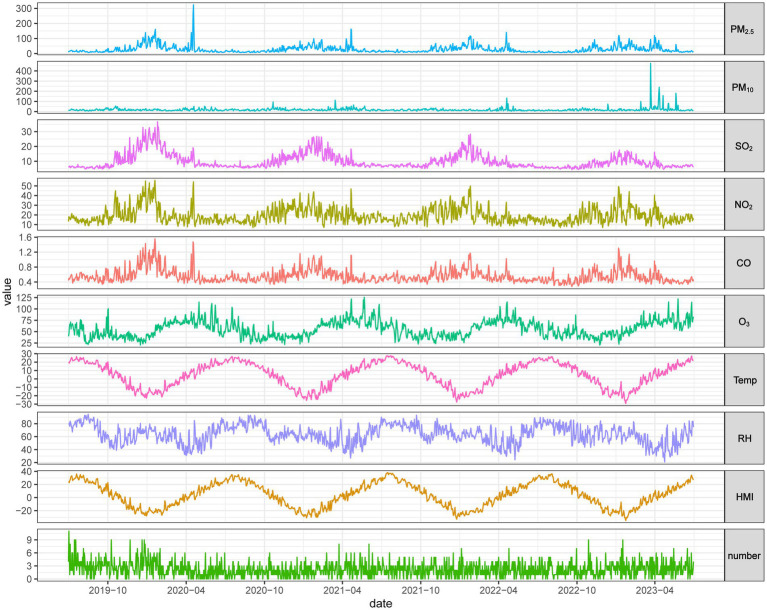
Time-series plots of air pollutants, temperature, relative humidity, Humidex, and PTB counts in Heilongjiang Province, 2019–2023.

The distribution of daily singleton PTB cases, meteorological factors, and air pollutants in Heilongjiang Province is shown in Table 1. During the study period from July 1, 2019, to June 30, 2023, a total of 3,267 PTB cases were included. Among them, the number of male PTB cases (*n* = 1,684) was higher than that of females (*n* = 1,583), and most mothers were under 35 years old (*n* = 2,420). The highest number of PTB cases occurred at gestational weeks ≥32 (*n* = 2,578). There was no significant difference in the number of PTB cases between warm and cold seasons, with cases distributed nearly evenly (approximately 1:1). During the study period, the mean temperature in Heilongjiang Province was 4.62 °C, with the minimum temperature of −29.34 °C. The mean relative humidity was 63.7%, and the mean Humidex was 3.78 °C. The mean concentrations of PM_2.5_, PM_10_, NO_2_, SO_2_, CO, and O_3_ were 27.92 μg/m^3^, 46.55 μg/m^3^, 9.9 μg/m^3^, 19.27 μg/m^3^, 0.56 mg/m^3^, and 55.56 μg/m^3^, respectively. The temporal variation characteristics of daily PTB cases, meteorological factors, and air pollutants concentrations in Heilongjiang Province from 2019 to 2023 are shown in Figure 1, all exhibited certain seasonality in their fluctuations.

**Table 1:** Summary statistics of air pollutants, meteorological factors, and daily preterm birth.

Variables	Number (%)	Mean (SD)	Min	P5	P25	P50	P75	P95	Max
Total	3,267(100)	2.24 (1.68)	0	0	1	2	3	5	11
Baby’s gender									
Male	1,684(51.5)	1.15 (1.12)	0	0	0	1	2	3	6
Female	1,583(48.5)	1.08 (1.14)	0	0	0	1	2	3	7
Maternal age									
<35	2,420(74.1)	1.66 (1.38)	0	0	1	1	2	4	8
≥35	847(25.9)	0.58 (0.81)	0	0	0	0	1	2	8
Gestational weeks									
<32	689 (21.1)	0.47 (0.77)	0	0	0	0	1	2	6
≥32	2,578 (78.9)	1.76 (1.47)	0	0	1	1	3	5	9
Season									
Warm	1,614(49.4)	2.22 (1.68)	0	0	1	2	3	5	11
Cold	1,653(50.6)	2.26(1.68)	0	0	1	2	3	5	9
Air pollutants									
PM_2.5_(μg/m^3^)	/	27.92 (24.97)	5.78	8.21	12.6	18.8	35.3	75.5	323.35
PM_10_(μg/m^3^)	/	46.55 (34.67)	11.26	17	26	36.90	56.7	104	567.67
SO_2_(μg/m^3^)	/	9.9 (5.07)	4.34	5.47	6.45	7.93	11.7	20.9	36.62
NO_2_(μg/m^3^)	/	19.27 (8.13)	6.32	10	13.7	17.1	22.8	35	55.8
CO(mg/m^3^)	/	0.56 (0.18)	0.28	0.37	0.44	0.51	0.63	0.92	1.56
O_3_(μg/m^3^)	/	55.56 (18.2)	20.47	30.5	40.7	53.7	68.5	86.5	125.74
Meteorological factors									
Temperature(°C)	/	4.62(14.77)	−29.34	−20.3	−8.39	6.82	18	24.1	27.59
Relative humidity (%)	/	63.7 (14.15)	20.75	38	54	64.5	74.5	85	94.65
Humidex(°C)	/	3.78(18.65)	−35	−25	−13	4	20	31	38

**Figure 2:**
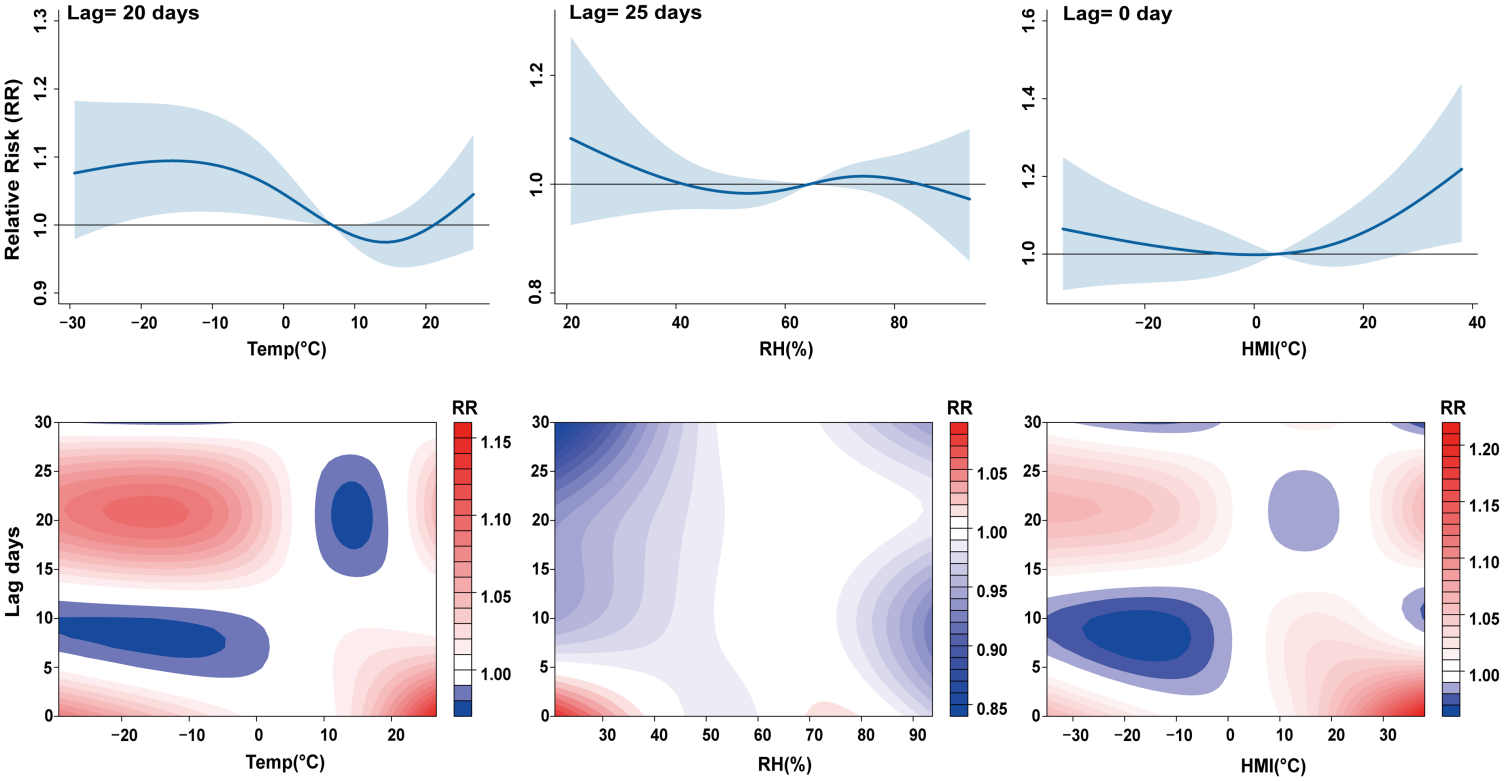
The exposure response relationship and contour diagrams between daily average temperature, relative humidity, Humidex, and PTB risk. RR, relative risk. Lag days (day).

**Figure 3:**
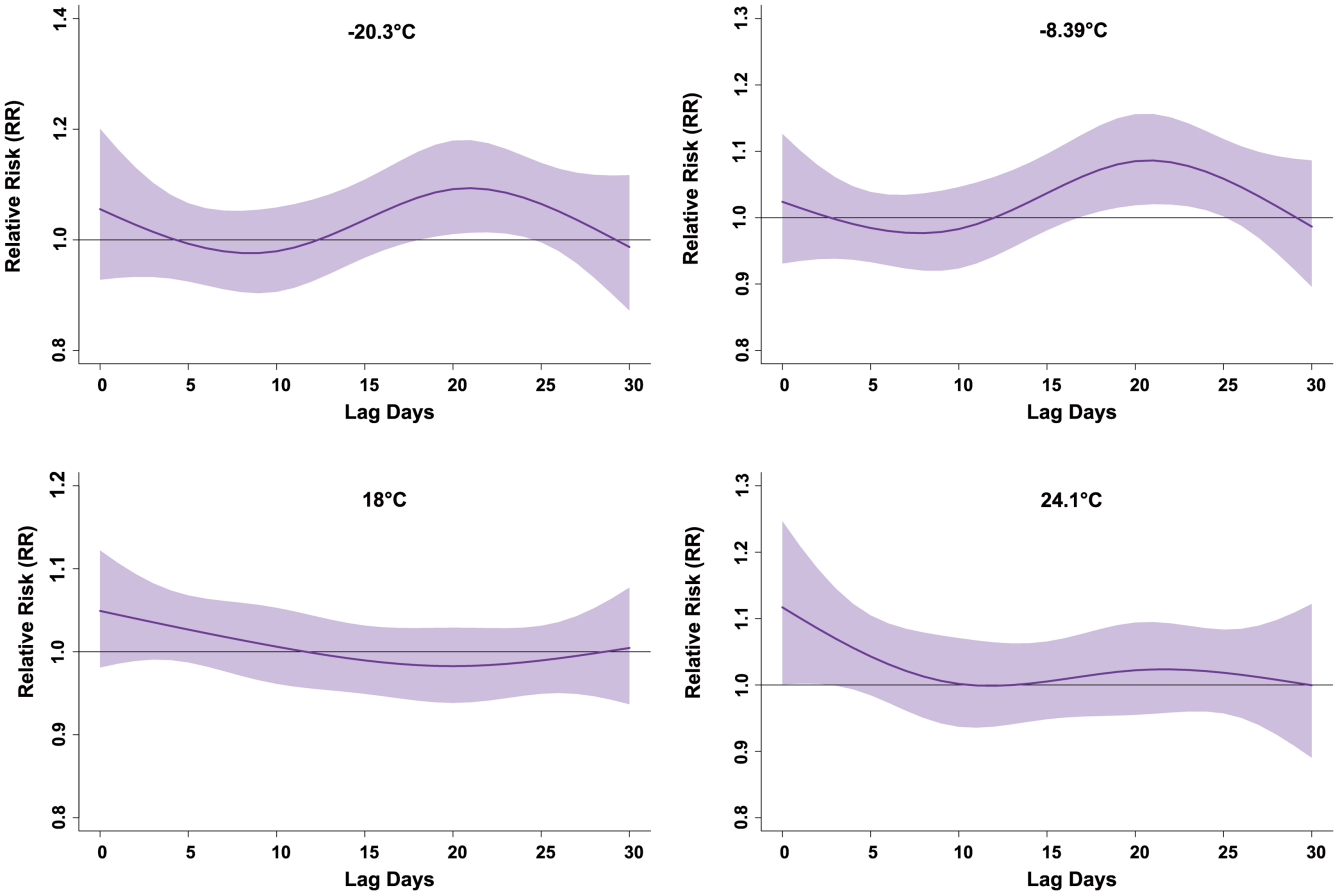
RR lag plot under extremely temperature distribution. This model is adjusted based on relative humidity, long-term trends and seasonality, DOW, and Holidays. All numbers are based on 6.82 °C as a reference. RR, relative risk. Lag days (day).

Figure 2 shows the exposure response and contour diagrams of temperature, relative humidity, and Humidex on PTB risk. The exposure response relationship showed that as temperature increased, the influence of temperature gradually weakened, and as relative humidity increased, its influence similarly decreased. In contrast, with the increase in Humidex, the risk of PTB increased. The contour diagram showed that as the lag days increased, the impact of temperature on PTB also increased, while the impact of Humidex weakened. Figure 3 shows the exposure response relationship for temperatures of −20.3, −8.39, 18, and 24.1 °C, corresponding to the 5, 25, 75, and 95th percentiles of the temperature distribution, respectively, compared with the median. With −20.3 °C and −8.39 °C, the risk of PTB increased, and the single-day lag effect reached its peak around the lag day 20, with RR = 1.09 (95% CI: 1.01–1.18) and RR = 1.09 (95% CI: 1.02–1.16), respectively. As the lag days continued to increase, the impact of extremely low temperatures and low temperatures weakened. The effect of high temperature (18 °C) on PTB was not statistically significant. During the lag period of 0–4 days, the single-day lag effect increased the risk of PTB at extremely high temperatures (24.1 °C), with RR = 1.12 (95% CI: 1.00–1.25). The cumulative lag effect of extremely high temperatures on the risk of PTB was significant from lag0–0 to lag0–6 days, showing an upward trend. With the maximum effect value occurring between lag days 0–6 days, RR = 1.62 (95% CI: 1.00–2.63) (Supplementary Figure S3). Overall, these findings indicate that exposure to extremely low, low temperatures was associated with an increased risk of PTB. But the result of extremely high temperatures may have borderline significance. This only provided preliminary evidence of potential association.

**Figure 4:**
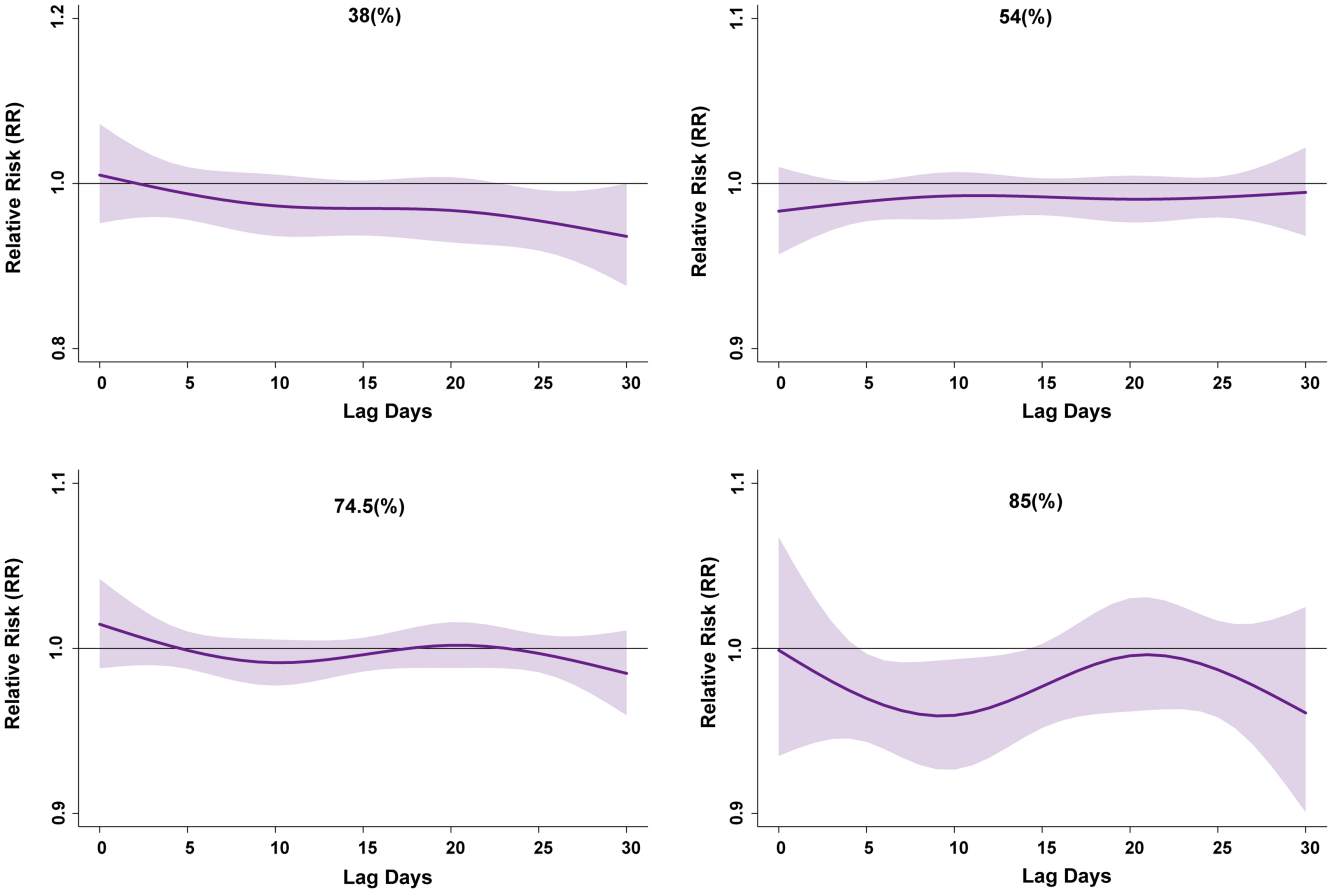
RR lag plot under extremely relative humidity distribution. This model is adjusted based on temperature, long-term trends and seasonality, DOW, and Holidays. All numbers are based on 64.5 (%) as a reference. RR, relative risk. Lag days (day).

Figure 4 shows the exposure response relationship of relative humidity corresponding to the 5, 25, 75, and 95th percentiles of distribution, compared with the median. The effects of 54 and 74.5% relative humidity on PTB were not statistically significant. With 38%, the single-day lag effect lasted for 4 days, and the risk of PTB was reduced, with RR = 0.95 (95% CI: 0.92–0.99) at lag day 25. With 85%, the single-day lag effect lasted for 6 days, and the risk of PTB was also reduced, with RR = 0.97 (95% CI: 0.94–0.99) at lag day 6. The cumulative lag effect of relative humidity on PTB risk was significant after approximately lag0–15 days, persisting until lag0–30 days. With 38%, the cumulative lag effect lasted for 4 days, and the risk of PTB was reduced, with RR = 0.48 (95% CI: 0.23–0.98) at lag0–27 days (Supplementary Figure S4). Although the results initially suggested that relative humidity seemed to be associated with a reduced risk of PTB, we consider that this finding may be part of the non-linear relationship mediated by temperature. Therefore, we introduced Humidex to comprehensively represent the effects of temperature and humidity on PTB.

**Figure 5:**
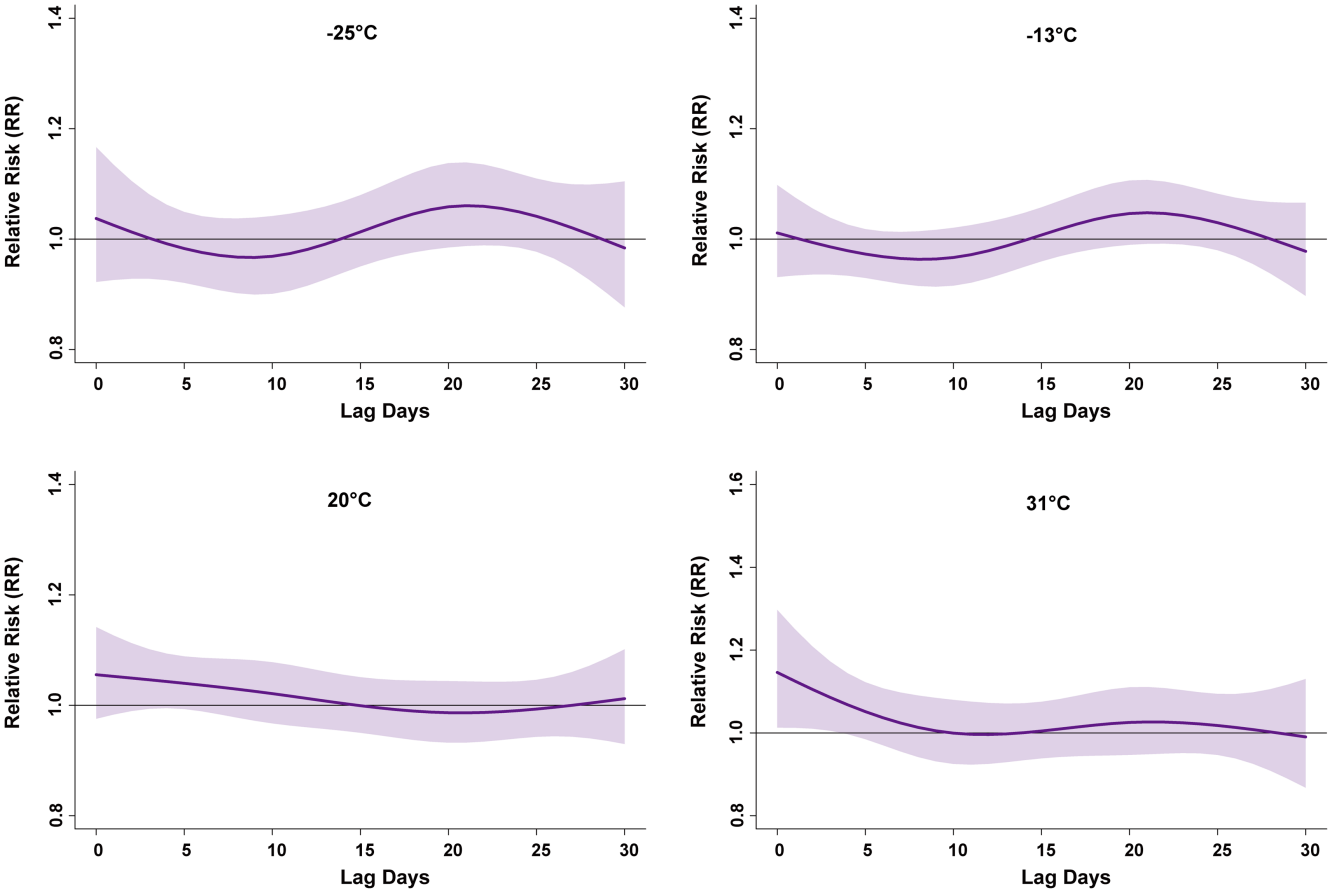
RR lag plot under extremely Humidex conditions. This model is adjusted based on long-term trends and seasonality, DOW, and Holidays. All numbers are based on 4 °C as a reference. RR, relative risk. Lag days (day).

Figure 5 shows the exposure response relationship for Humidex of −25, −13, 20, and 31 °C, corresponding to the 5, 25, 75, and 95th percentiles of the Humidex distribution, respectively, compared to the median. The effects of −25, −13, and 20 °C on PTB were not statistically significant. With 31 °C, the risk of PTB increased, the single-day lag effect lasted for 5 days, with RR = 1.15 (95% CI: 1.01–1.3) at lag day 0. The cumulative lag effect of extremely high Humidex on PTB risk was significant from lag0–0 to lag0–9 days and showed a decreasing trend, with the maximum effect value of RR = 1.15(95% CI: 1.01–1.3) (Supplementary Figure S5). Taken together, these results suggest that exposure to extremely high Humidex was associated with an increased risk of PTB.

**Figure 6:**
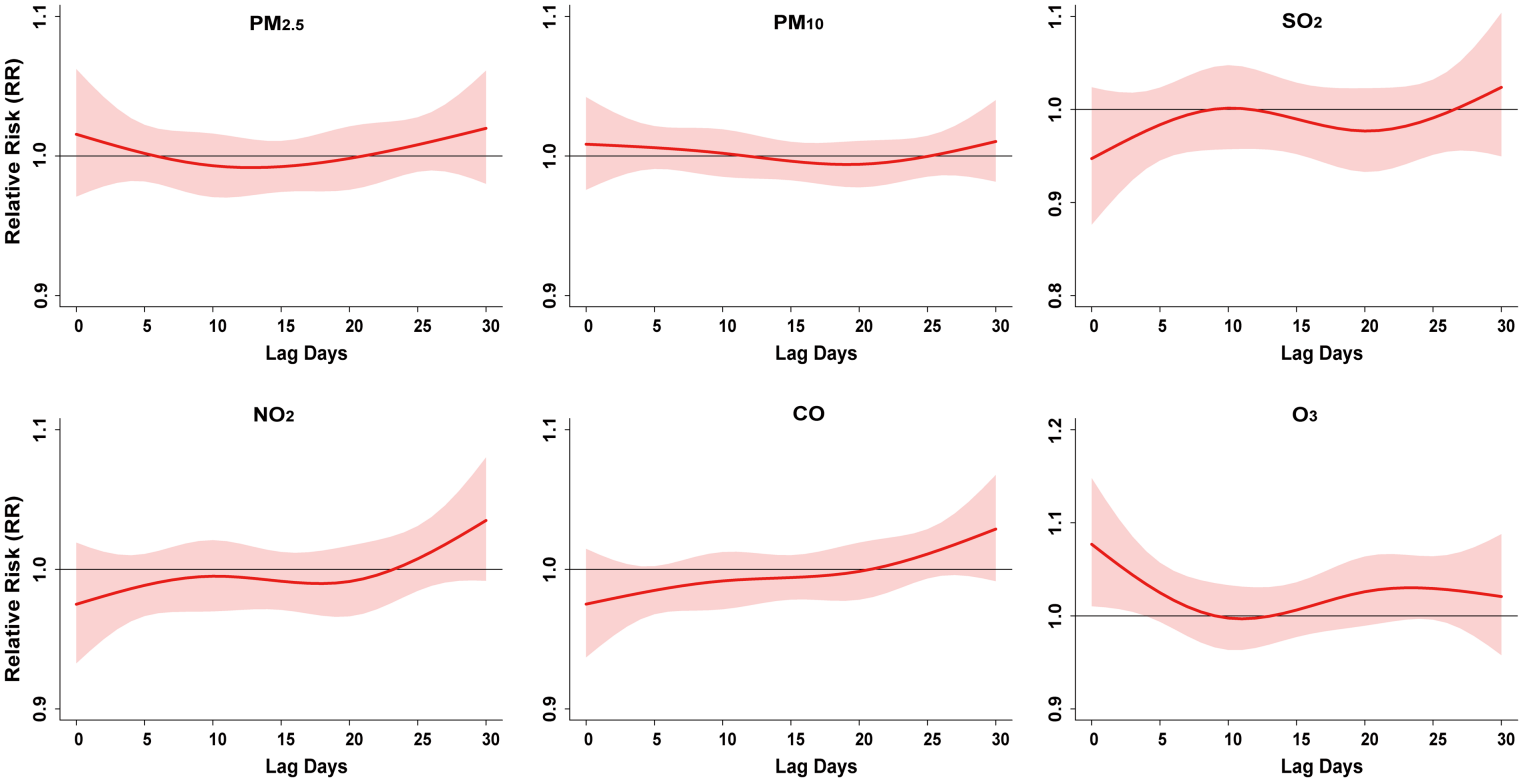
The single-day lag effect of an IQR increase in exposure to air pollutants on the RR of PTB; PM_2.5_, particulate matter <2.5 μm; PM_10_, particulate matter <10 μm; SO_2_, sulfur dioxide; NO2, nitrogen dioxide; CO, carbon monoxide; O_3_, ozone. RR, relative risk. Lag days (day).

Figure 6 and Supplementary Figure S8 show the single-day lag effect and the cumulative lag effects of an IQR increase in exposure to air pollutants on the risk of PTB. Apart from O_3_, no statistically significant associations were observed between other air pollutants and PTB risk. For O_3_, each IQR increase (27.8 μg/m^3^) was associated with an increased risk of PTB: the single-day lag effect lasted for 5 days, with the highest risk observed at lag day 0 (RR = 1.08, 95% CI: 1.01–1.15). Additionally, the cumulative lag effect of O_3_ exposure on PTB risk was significant, with the maximum effect observed at lag 0–8 days (RR = 1.38, 95% CI: 1.02–1.88).

Supplementary Figures S6, S7 show the exposure–response and contour diagrams of air pollutant exposure in relation to PTB risk. However, apart from O₃, there were no statistically significant associations between other air pollutants and PTB risk. As O₃ concentration increased, the risk of PTB also increased.

Supplementary Figures S9–S12 show the RR (95% CI) of PTB stratified by maternal age, gestational weeks, and season for different lag periods, which were broadly consistent with the overall results. The effect of temperature was greater among pregnant women younger than 35 years compared with those aged 35 years or older, similar to the results of relative humidity and Humidex, while the effect of ozone was the opposite. During the warm season, higher temperatures were associated with an increased risk of PTB, whereas in the cold season, higher temperatures were linked to a lower risk of PTB. The impact of Humidex was greater in the warm season than in the cold season. Pregnant women with gestational weeks greater than 32 weeks were more sensitive to temperature and ozone than those with gestational weeks less than 32 weeks, while relative humidity was the opposite. In the warm season, as the concentration of O_3_ increased, the risk of PTB also increased over a lag period spanning from 19 to 26 days, whereas no statistically significant association was observed between O_3_ concentration and PTB risk in the cold season.

Given that the correlation coefficient between SO_2_ and temperature stood at −0.7, SO_2_ was excluded from the dual pollutant model involving temperature to prevent multicollinearity (Supplementary Figure S2). We selected the lag days with the maximum effects of temperature, relative humidity, Humidex, and O_3_ from the single-pollutant model and constructed a dual pollutant model to verify model stability (Supplementary Figure S13). The associations of temperature, relative humidity, and O₃ with PTB remained stable and were consistent with the results of the single-pollutant models. Although the inclusion of other pollutants slightly affected Humidex, the results of CO remained stable. To further assess model robustness, we varied the degrees of freedom for the time variable from 6 to 8 and for relative humidity from 2 to 4 (Supplementary Table S1). The results indicate that, although there were fluctuations in the degrees of freedom, the consistency of the model output ensured the reliability of the results. We also selected different maximum lag periods and excluded cases of preeclampsia and gestational hypertension (Supplementary Table S2). The results reveal that the model with a maximum lag of 28 days closely resembles the main model, thereby reinforcing the alignment with previous research. Moreover, considering that pregnancy is a prolonged and intricate biological process, it is necessary to thoroughly investigate the lag effect. Thus, setting the maximum lag day at 30 days is appropriate to offer a balanced and all-encompassing viewpoint for our analysis. After excluding cases of preeclampsia and gestational hypertension, the model results were consistent with the main model, demonstrating the robustness of our model.

## 4 Discussion

PTB is defined as the birth of a live infant before 37 completed weeks of gestation. PTB is one of the main causes of neonatal mortality and childhood health. This study is the first to explore the nonlinear effects between meteorological factors and air pollutants in cold regions on PTB risk through time series analysis. Our findings indicate that: 1. Exposure to extreme temperatures was significantly associated with an increased risk of PTB. 2. An extremely high Humidex was associated with an increased risk of PTB.3. An increase in ozone concentration was associated with an increased risk of PTB.

The research conclusion on the statistically significant association between extremely high temperatures and PTB is highly consistent with the results of two recent review studies, both of which reported a significant association between high temperatures and PTB (23, 24). The biological mechanisms underlying the association between environmental temperature and PTB are not yet clear. Exposure to high temperatures can cause maternal dehydration and reduced uterine blood flow, which may trigger uterine contractions and initiate labor (25). In addition, animal studies have demonstrated that heat exposure may increase the secretion of pro-inflammatory cytokines and oxytocin to induce childbirth (26, 27). Heat exposure may increase the risk of prenatal membrane rupture and infection, which may be associated with an increased risk of PTB (28).

Currently, research on the association between low temperature and PTB remains inconsistent. A study covering 132 cities in China reported that exposure to cold was associated with a reduced risk of PTB (29). In contrast, a time-series analysis of a large 10-year birth cohort in Rome found no statistically significant association between extreme cold and PTB (30). Conversely, a large-scale and population-based epidemiological study in China indicated that low temperatures appear to be a risk factor for PTB (31), which is consistent with our findings. This may be explained by the extremely low winter temperatures in Heilongjiang Province, which may have adverse effects on pregnant women and fetuses through various mechanisms. Extremely low temperatures may increase the risk of PTB. The cold thermoregulatory response may involve increased blood viscosity and vasoconstriction, as well as elevated hormone levels and accelerated birth time (32).

Few studies have investigated the effect of relative humidity on PTB. A small sample study in Romania analyzed the impact of atmospheric conditions on PTB and found no significant association between humidity and PTB (33). In contrast, a large population-based cohort study in Yunnan, China, found that low humidity was a protective factor for PTB. However, high humidity was a risk factor for PTB (34). Although our preliminary findings initially point to an association between relative humidity and a decreased risk of PTB, we believe that temperature may play a significant confounding role. To address this, we introduced the Humidex, a comprehensive indicator first proposed by Masterton and Richardson of the Canadian Bureau of Meteorology. As a comprehensive indicator that quantifies both temperature and humidity, it helps to more comprehensively evaluate the interaction between these environmental factors and human comfort, taking into account both temperature and humidity (35). Our research results on an extremely high humidex are consistent with previous studies, indicating that extremely high humidex increases the risk of PTB (9). Although the precise mechanisms through which temperature and humidity affect human health remain incompletely understood, current evidence indicates a non-linear association between these factors and health outcomes, suggesting that their combined influence may exceed the sum of their individual effects (36). Even within the specific temperature range of our study, Humidex provided a more accurate reflection of the association between combined thermal exposure and PTB risk in cold regions.

Two reviews on the relationship between O_3_ and PTB suggested that O_3_ exposure increases the risk of PTB, which is consistent with our findings (37, 38). For example, a study using 34,122 singleton live birth records from Beijing between 2016 and 2019 found that each 10 μg/m^3^ increase in O₃ exposure was associated with a 3.9% increase in PTB risk (95% CI: 0.6–7.3%) and identified specific pregnancy windows of heightened susceptibility (39). Exposure to ozone can trigger coagulation processes and has the potential to induce vascular complications, such as placental abruption, which may ultimately result in premature birth. Additionally, ozone exposure is linked to the activation of the hypothalamic–pituitary–adrenal (HPA) axis, a system responsible for cortisol secretion. Cortisol, in turn, may exacerbate oxidative stress following pollutant exposure by boosting the production of reactive oxygen species (40, 41). Animal model studies have also shown that O_3_ can cause oxidative stress, affecting maternal circulation and cardiovascular health, including increased uterine artery resistance index and placental transcriptional changes (42, 43). It may also impair fetal oxygen and nutrient transport by inducing oxidative stress, inflammation, and hemodynamic changes (44). The research results in London, UK, suggested that exposure to higher levels of O_3_ and PM_2.5_ during pregnancy may increase the risk of PTB (45). The research results of O_3_ are consistent with our conclusion, while PM_2.5_ is different. The birth cohort study in Chongqing, China, showed no statistically significant associations for SO_2_ and NO_2_ during each pregnancy period, which is consistent with our research results, but found that maternal exposure to high levels of PM_2.5_, PM_10_, and CO during pregnancy may increase the risk of PTB is inconsistent with our research results (46). By contrast, a nationwide study in Sweden found that an increase in ozone exposure is associated with an increased risk of PTB, while PM_2.5_ and PM_10_ are not significantly correlated with PTB, which is consistent with our research findings (40). Likewise, a retrospective cohort study in Guangzhou did not observe any association between SO₂ exposure and PTB risk (47). Our results on pollutant associations are contrary to those reported in other major Chinese cities, such as Beijing and Chongqing—a discrepancy likely attributable to the lower ambient pollutant concentrations in Heilongjiang Province. The average concentrations of PM_2.5_, PM_10_, and CO in Chongqing are 41.62 μg/m^3^, 66.39 μg/m^3^, and 1.02 mg/m^3^, while the average concentration of PM_2.5_ in Beijing is 70.4 μg/m^3^. In contrast, the average concentrations of PM_2.5_, PM_10_, and CO in Heilongjiang Province are 27.92 μg/m^3^, 46.55 μg/m^3^, and 0.56 mg/m^3^, which are much lower than those in areas with higher industrialization levels. These substantial differences in exposure metrics—particularly in regions with higher industrial activity—may fundamentally alter the observed pollutant-health associations (10, 46). Taken together, our results suggest that O₃ was associated with an increased risk of PTB, while the associations between other air pollutants and PTB warrant further investigation.

Our research has several potential limitations. First, the meteorological data were derived from the average of 54 monitoring stations in Heilongjiang Province, which may not accurately represent individual exposure and may have potential spatial heterogeneity; therefore, causal conclusions regarding individual-level associations cannot be established. Second, this study utilized ecological research methods, which limit the inference of causal relationships between exposure factors and outcomes. As a result, it is difficult to completely avoid ecological fallacies, so caution must be taken when generalizing research results to other regions. Compared with other multi-regional and multicenter studies, the geographical location and population limitations of this study may limit the generalizability of the research results. This study obtained data from two hospitals in Heilongjiang Province. Although both hospitals have comprehensive coverage in Heilongjiang Province, a large number of patient visits, strong representativeness of disease sources, and reliable case data, there are certain limitations in extending the results to other countries or regions. Third, other confounding factors such as genetic factors, dietary habits, maternal education, smoking, infection, and occupational exposure may not have been considered in this study. The next step of research should include these confounding factors to determine their impact on PTB. Fourth, potential misclassification without considering gestational age, unadjusted seasonal conception, potential co-pollutant confounding, and temporal autocorrelation may affect risk estimation. More research is needed in the future on the relationship between meteorological factors and air pollutants, as well as PTB. Future studies also could integrate behavioral and psychosocial variables, such as health literacy or anxiety related to environmental hazards, to better model individual-level vulnerability and resilience (48, 49).

## 5 Conclusion

Our research findings indicated that extreme temperatures, an extremely high Humidex, and increased ozone concentrations are associated with an increased risk of preterm birth. These results provide further evidence for the association between meteorological factors, air pollutants, and preterm birth risk, and provide more theoretical basis for short-term early warning of preterm birth in cold regions and long-term emission reduction strategies for air pollutants.

## Data Availability

The datasets generated and analyzed during the current study are not publicly available because it is not an open data source, but are available from the corresponding author on reasonable request.

## Footnotes

1 http://www.csgnetwork.com/
