# Supplementary material for: Ambient temperature and ozone exposure increases the risk of preterm birth in Northeast China: a time series analysis
Source: Front Public Health. 2025 Dec 5;13:1712825. doi: 10.3389/fpubh.2025.1712825 (PMC12714875; doi:10.3389/fpubh.2025.1712825)
Supplement: Supplementary file 1 [file Data_Sheet_1.PDF]

Supplementary Material

This file includes:

Table S1. The relative risk with 95% CI in preterm birth is associated with temperature, relative humidity, Humidex, and ozone, according to different degrees of freedom (df) for calendar time and relative humidity
Table S2. The relative risk with 95% CI in preterm birth is associated with temperature, relative humidity, Humidex, and ozone, according to different maximum lag days and excluding cases of preeclampsia and gestational hypertension
Fig. S1. Roadmap of case selection
Fig. S2. Analysis results of the correlation between air pollutants and meteorological factors
Fig. S3. The lag-specific cumulative RR and 95% CI under extreme temperature exposure distribution
Fig. S4. The lag-specific cumulative RR and 95% CI under extreme relative humidity exposure distribution
Fig. S5. The lag-specific cumulative RR and 95% CI under extreme Humidex exposure distribution
Fig. S6. The contour diagrams between air pollutants and PTB risk
Fig. S7. The exposure-response relationship between air pollutants and PTB risk
Fig. S8. The cumulative lag effect of an IQR increase in exposure to air pollutants on the RR of PTB
Fig. S9: Lag-specific relative risk of temperature and preterm birth (95% CI) stratified by maternal age, gestational weeks, and season
Fig. S10. Lag-specific relative risk of relative humidity and preterm birth (95% CI) stratified by maternal age, gestational weeks, and season
Fig. S11. Lag-specific relative risk of Humidex and preterm birth (95% CI) stratified by maternal age, gestational weeks, and season
Fig. S12. Lag-specific relative risk of an IQR increase in ozone exposure and preterm birth (95% CI) stratified by maternal age, gestational weeks, and season
Fig. S13. The relative risk (with 95% CI) of PTB associated with temperature, relative humidity, Humidex, and ozone under multiple pollutant models

Table S1. The relative risk with 95% CI in preterm birth is associated with temperature, relative humidity, Humidex, and ozone, according to different degrees of freedom (df) for calendar time and relative humidity

	Df	Temperature [a]	Relative humidity [b]	Humidex [c]	O3 [d]
Time	6	1.06 (1.00-1.12)*	0.96 (0.93-0.99)*	1.11(0.99-1.26)	1.08 (1.01-1.15)*
	7	1.09 (1.02-1.16)*	0.95 (0.92-0.99)*	1.15(1.01-1.30)*	1.08 (1.01-1.15)*
	8	1.06 (1.00-1.12)*	0.97 (0.93-1.01)	1.10(0.97-1.25)	1.08 (1.01-1.15)*
Relative humidity	2	1.08 (1.01-1.15)*	/	/	1.08 (1.01-1.15)*
	3	1.09 (1.02-1.16)*	/	/	1.08 (1.01-1.15)*
	4	1.09 (1.02-1.16)*	/	/	1.08 (1.01-1.15)*

Note: a: The associations of preterm birth with low temperature (-8.39 °C) compared to the referent temperature (6.82 °C) have different degrees of freedom (df) for calendar time and relative humidity. b: The associations of preterm birth with low relative humidity (38%) compared to the referent relative humidity (64.5%) have different degrees of freedom (df) for calendar time. c: The associations of preterm birth with high humidex (31 °C) compared to the referent humidex (4 °C) have different degrees of freedom (df) for calendar time. d: The associations of preterm birth with an IQR increase in ozone exposure have different degrees of freedom (df) for calendar time and relative humidity. * Statistically significant.

Table S2. The relative risk with 95% CI in preterm birth is associated with temperature, relative humidity, Humidex, and ozone, according to different maximum lag days and excluding cases of preeclampsia and gestational hypertension

		Temperature [a]	Relative humidity [b]	Humidex [c]	O3 [d]
Lag day	7	1.04 (0.91-1.18)	0.97 (0.91-1.05)	1.13 (0.99-1.30)	1.01 (0.88-1.16)
	14	1.03 (0.93-1.13)	0.97 (0.93-1.02)	1.13 (0.99-1.28)	1.06 (0.96-1.17)
	21	1.09 (0.98-1.22)	0.96 (0.92-1.00)*	1.11 (1.01-1.21)*	1.06 (1.00-1.12)*
	28	1.08 (1.02-1.15)*	0.97 (0.93-1.00)*	1.10 (1.01-1.20)*	1.08 (1.01-1.15)*
Exclude preeclampsia and gestational hypertension (n=277)		1.08 (1.01-1.16)*	0.95 (0.91-0.99)*	1.16 (1.02-1.32)*	1.05 (1.00-1.11)*

Note: a: The associations of preterm birth with low temperature (-8.39 °C) compared to the referent temperature (6.82 °C) have different degrees of freedom (df) for calendar time and relative humidity. b: The associations of preterm birth with low relative humidity (38%) compared to the referent relative humidity (64.5%) have different degrees of freedom (df) for calendar time. c: The associations of preterm birth with high humidex (31 °C) compared to the referent humidex (4 °C) have different degrees of freedom (df) for calendar time. d: The associations of preterm birth with an IQR increase in ozone exposure have different degrees of freedom (df) for calendar time and relative humidity. * Statistically significant.

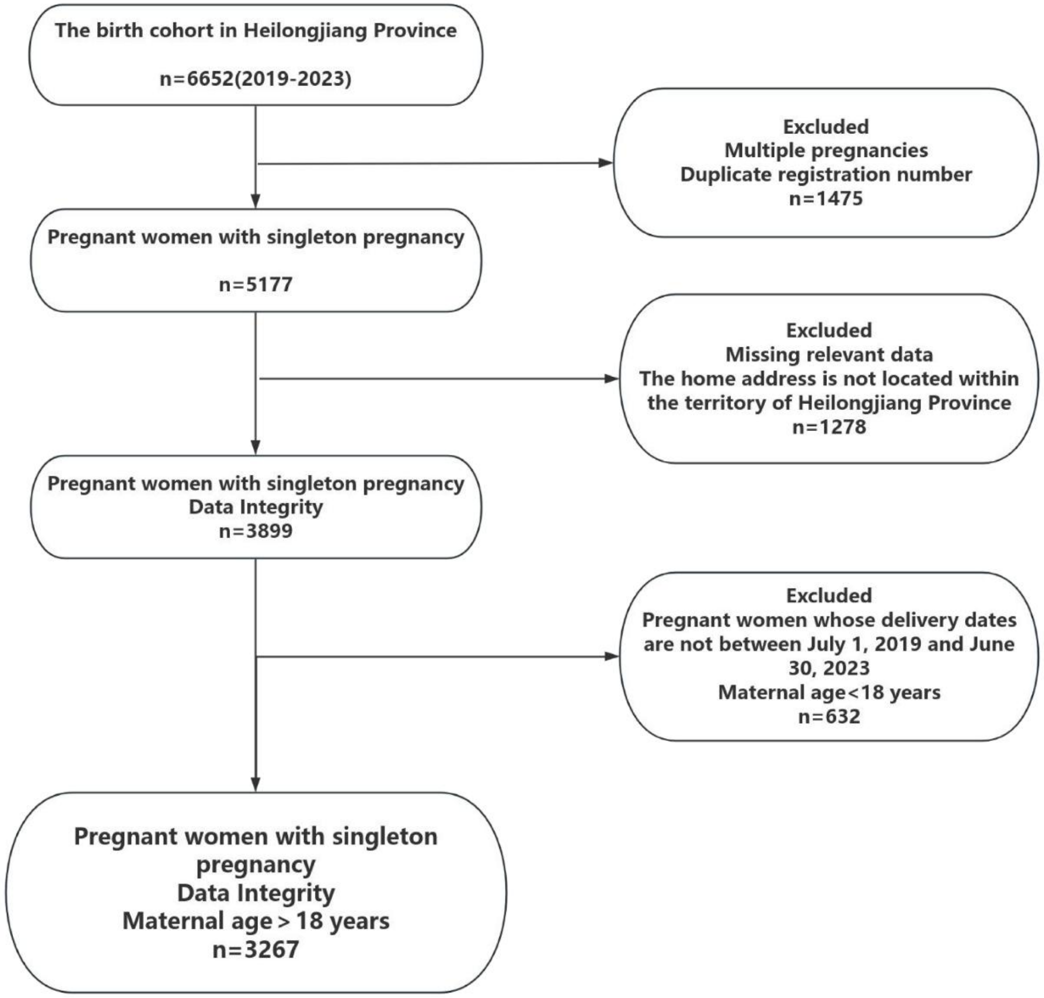

Fig. S1. Roadmap of case selection

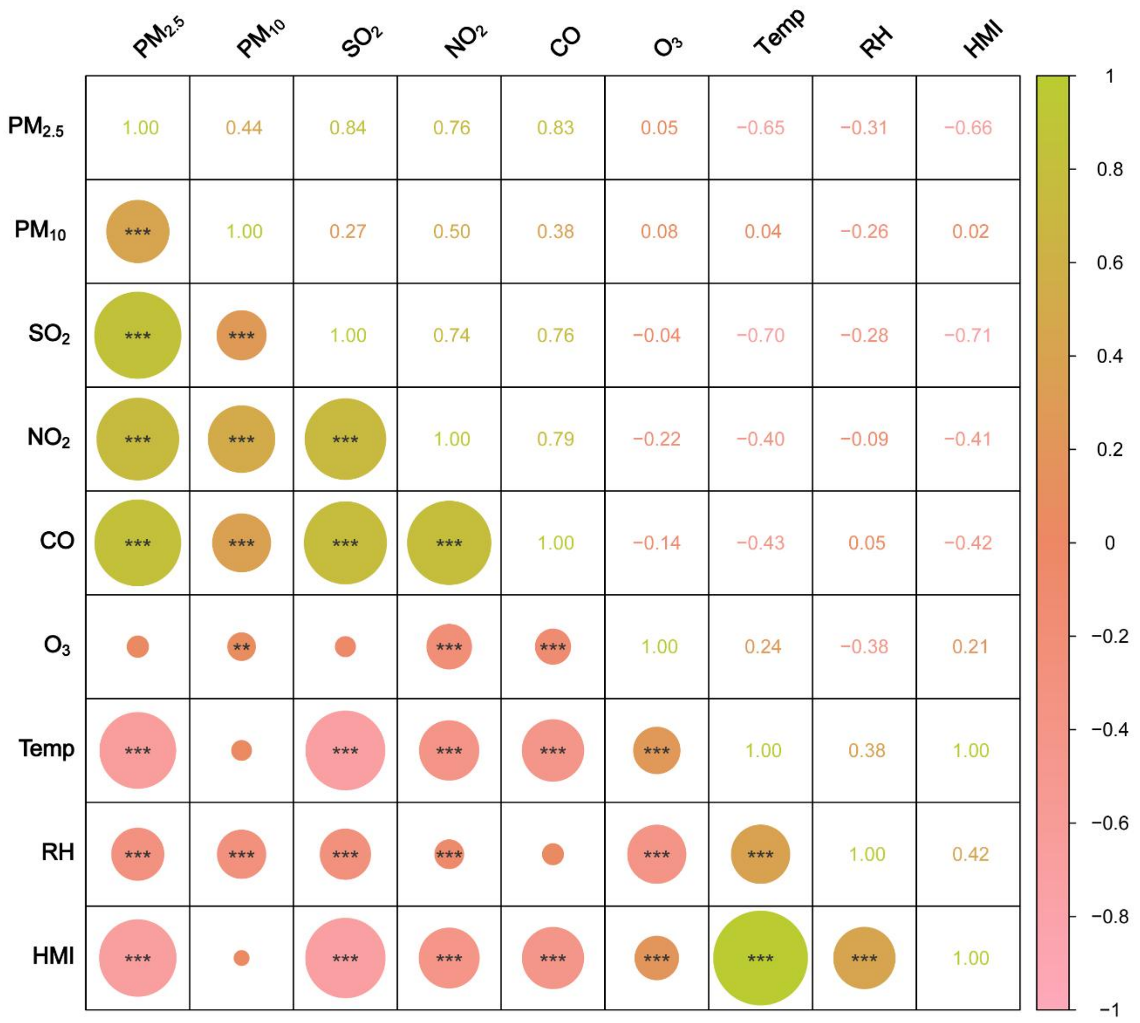

Fig. S2. Analysis results of the correlation between air pollutants and meteorological factors

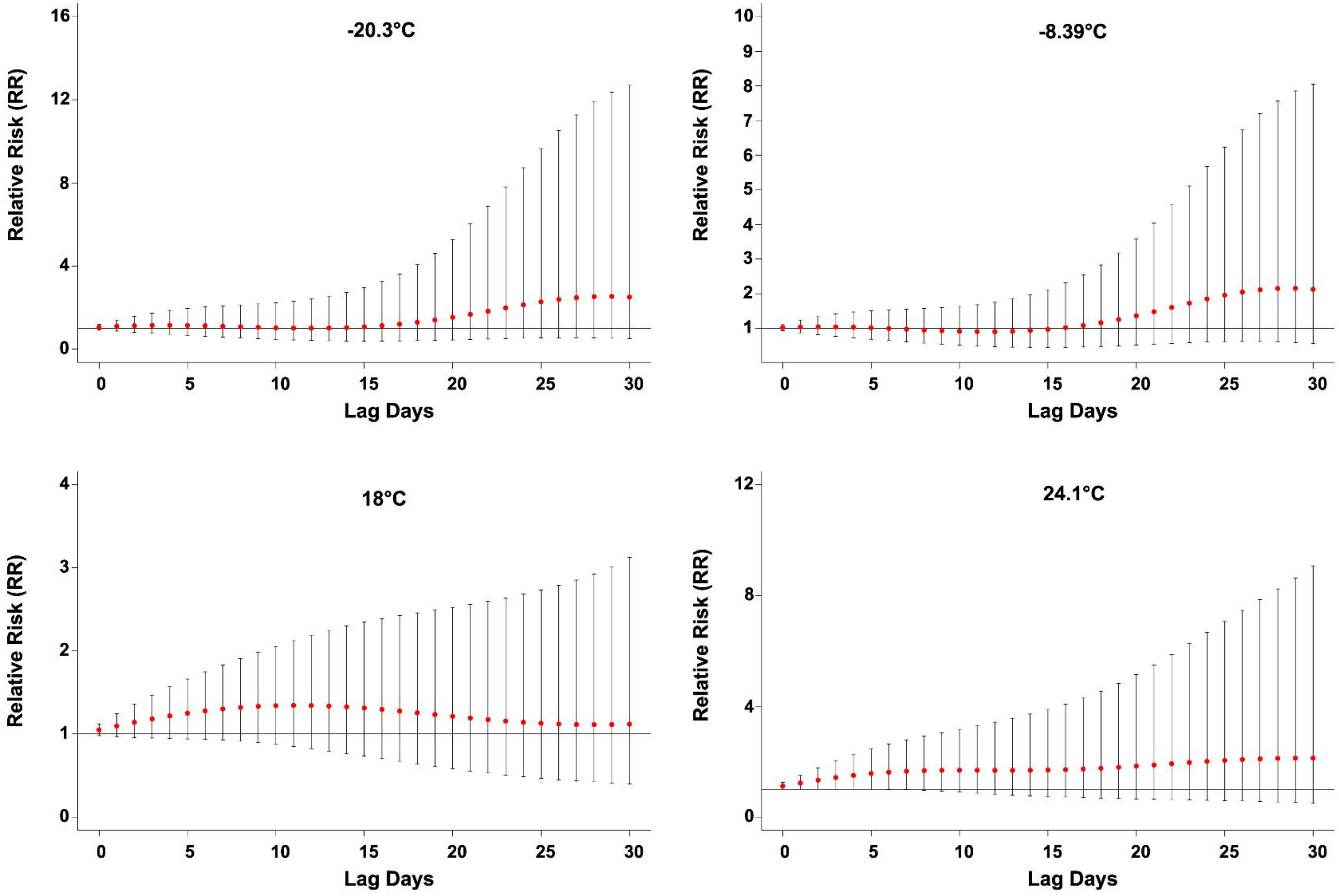

Fig. S3. The lag-specific cumulative RR and 95% CI under extreme temperature exposure distribution. RR, relative risk. Lag days (day)

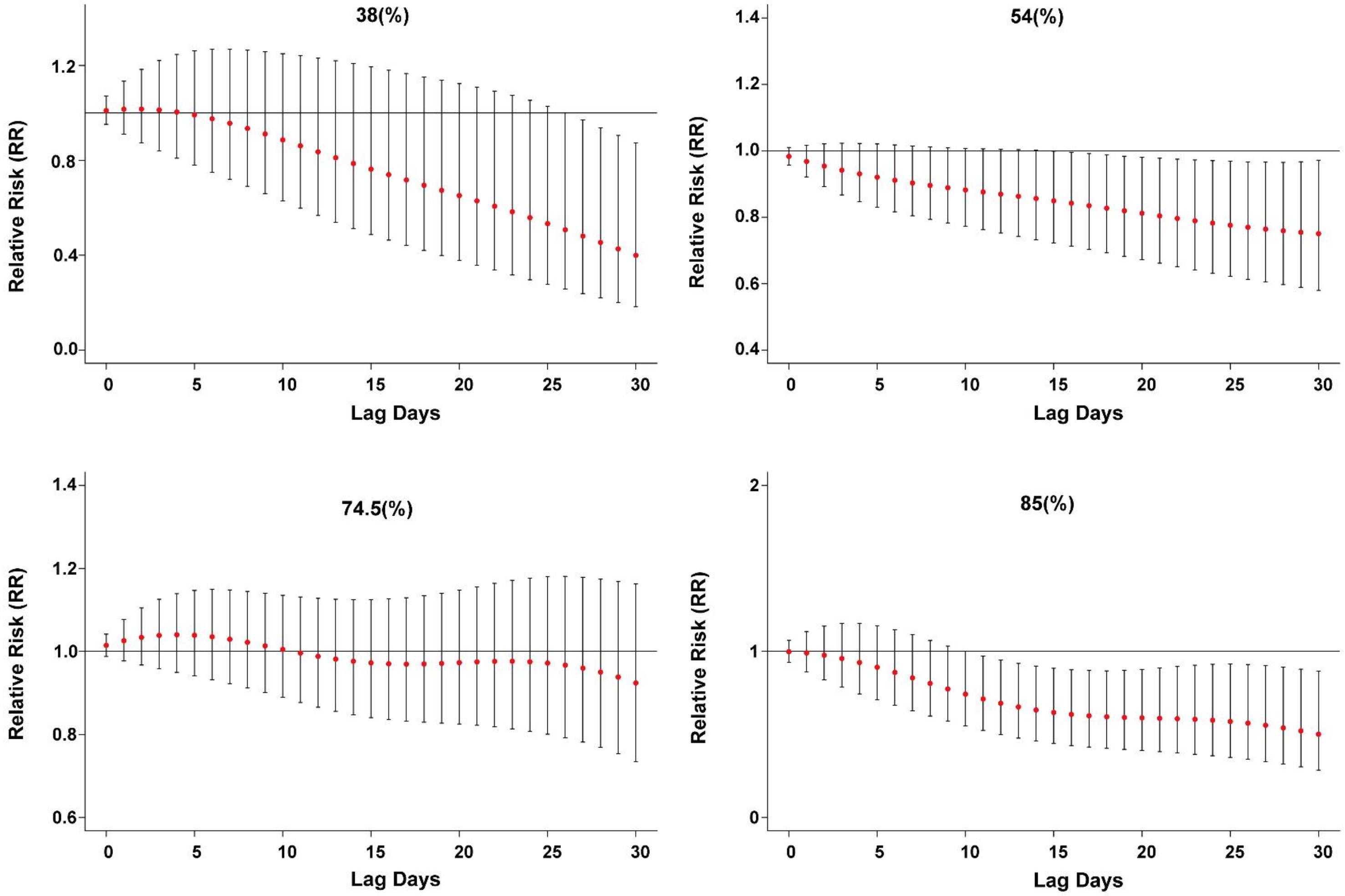

Fig. S4. The lag-specific cumulative RR and 95% CI under extreme relative humidity exposure distribution. RR, relative risk. Lag days (day)

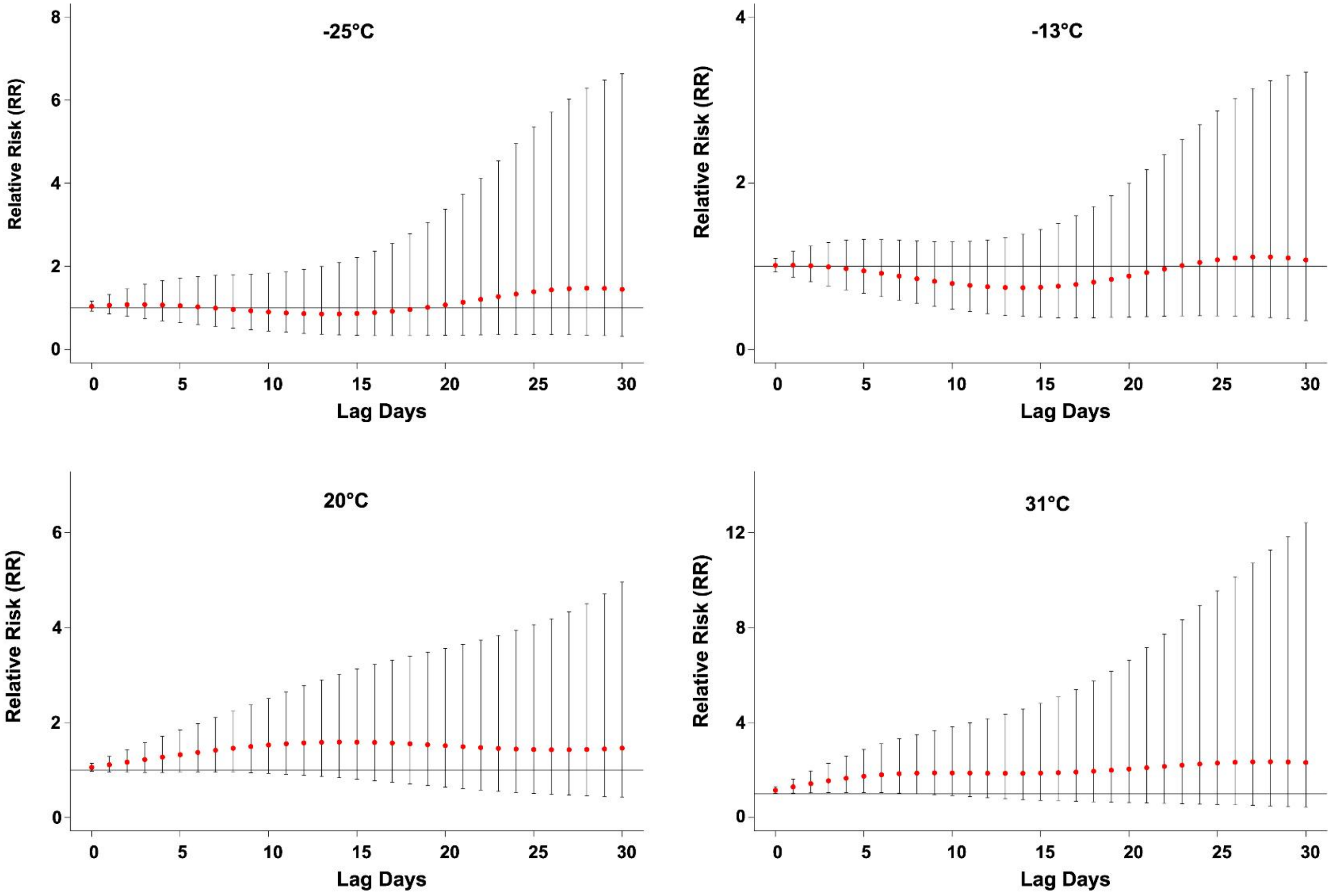

Fig. S5. The lag-specific cumulative RR and 95% CI under extreme Humidex exposure distribution. RR, relative risk. Lag days (day)

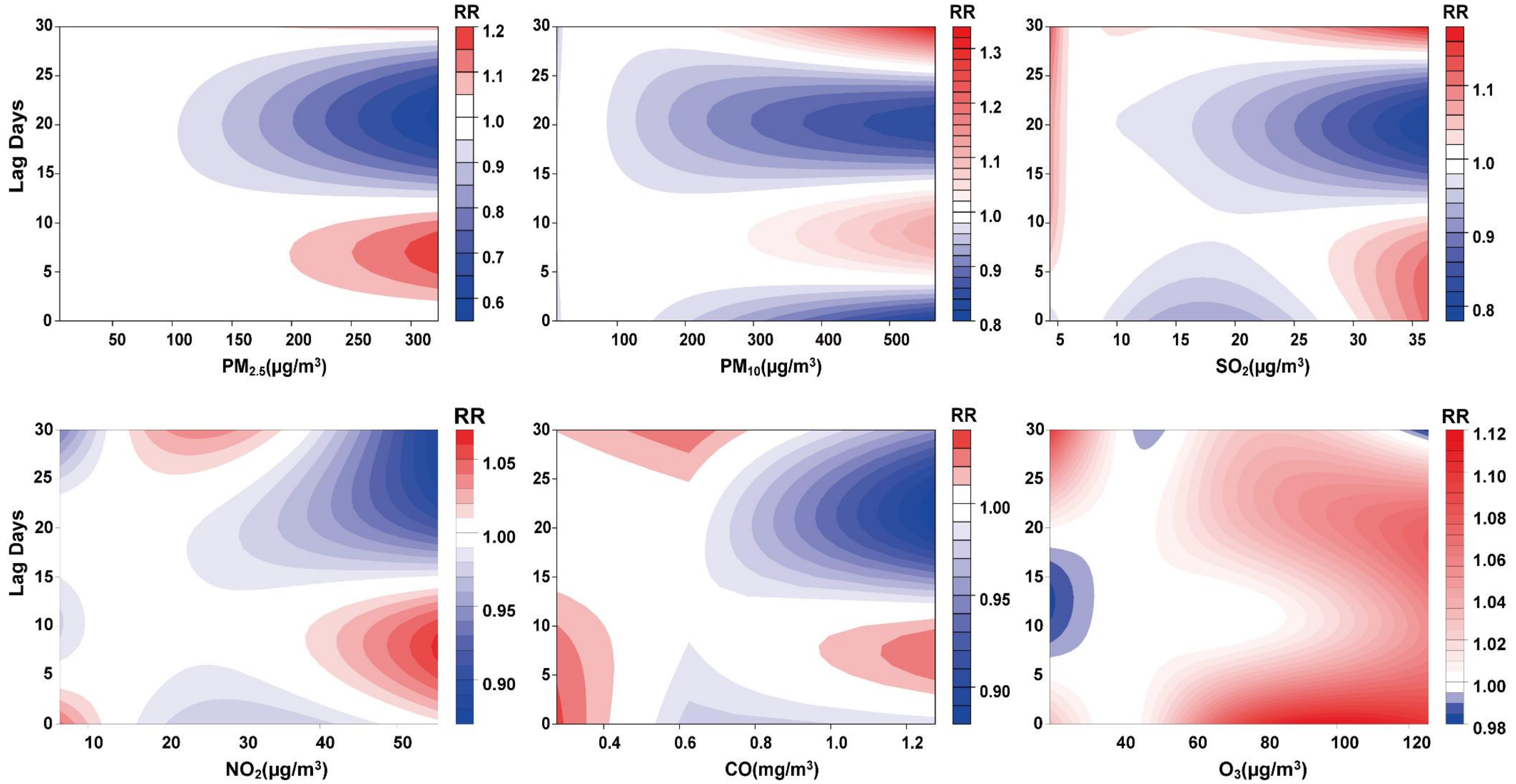

Fig. S6. The contour diagrams between air pollutants and PTB risk. RR, relative risk. Lag days (day)

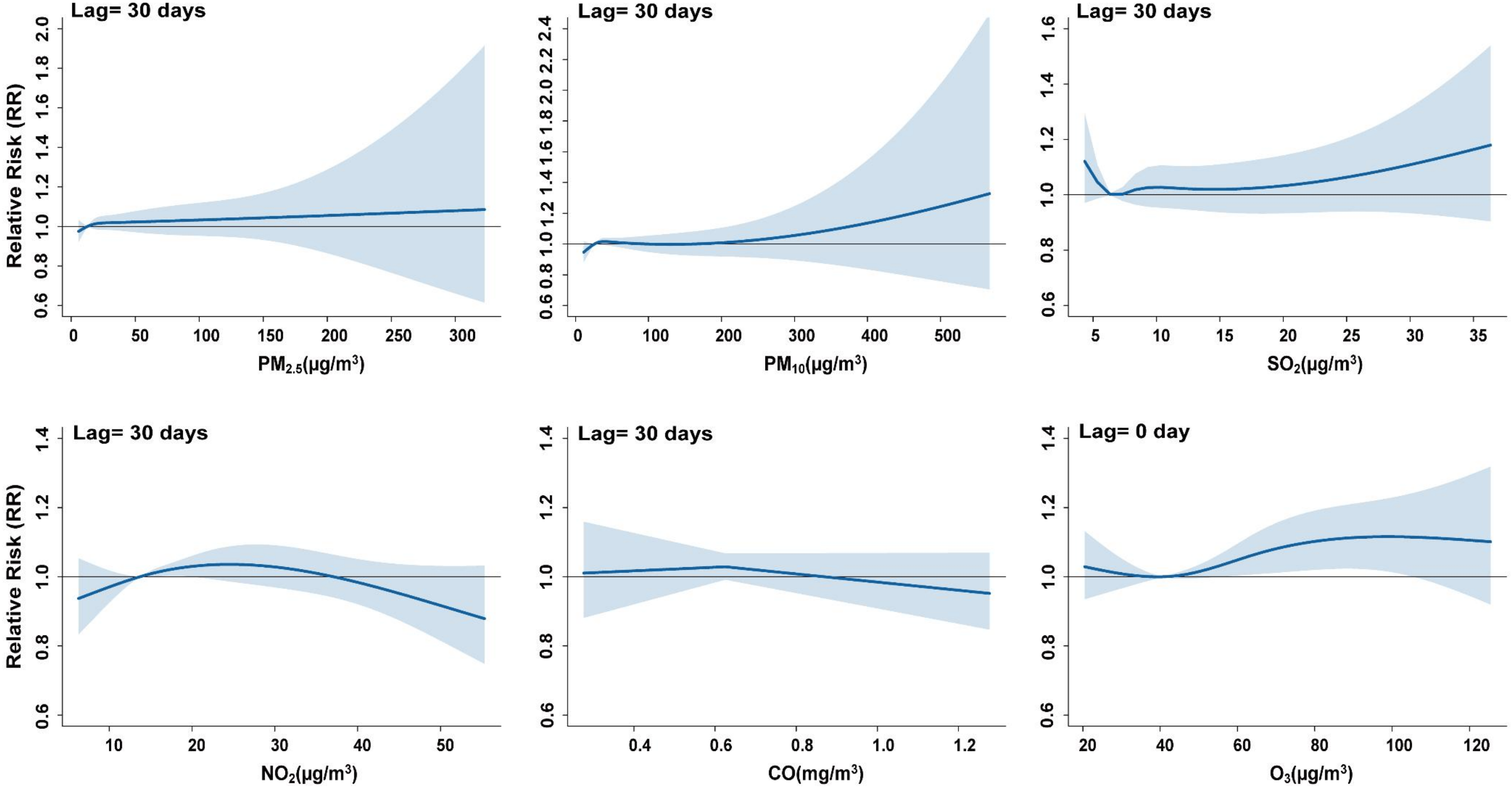

Fig. S7. The exposure-response relationship between air pollutants and PTB risk. RR, relative risk. Lag days (day)

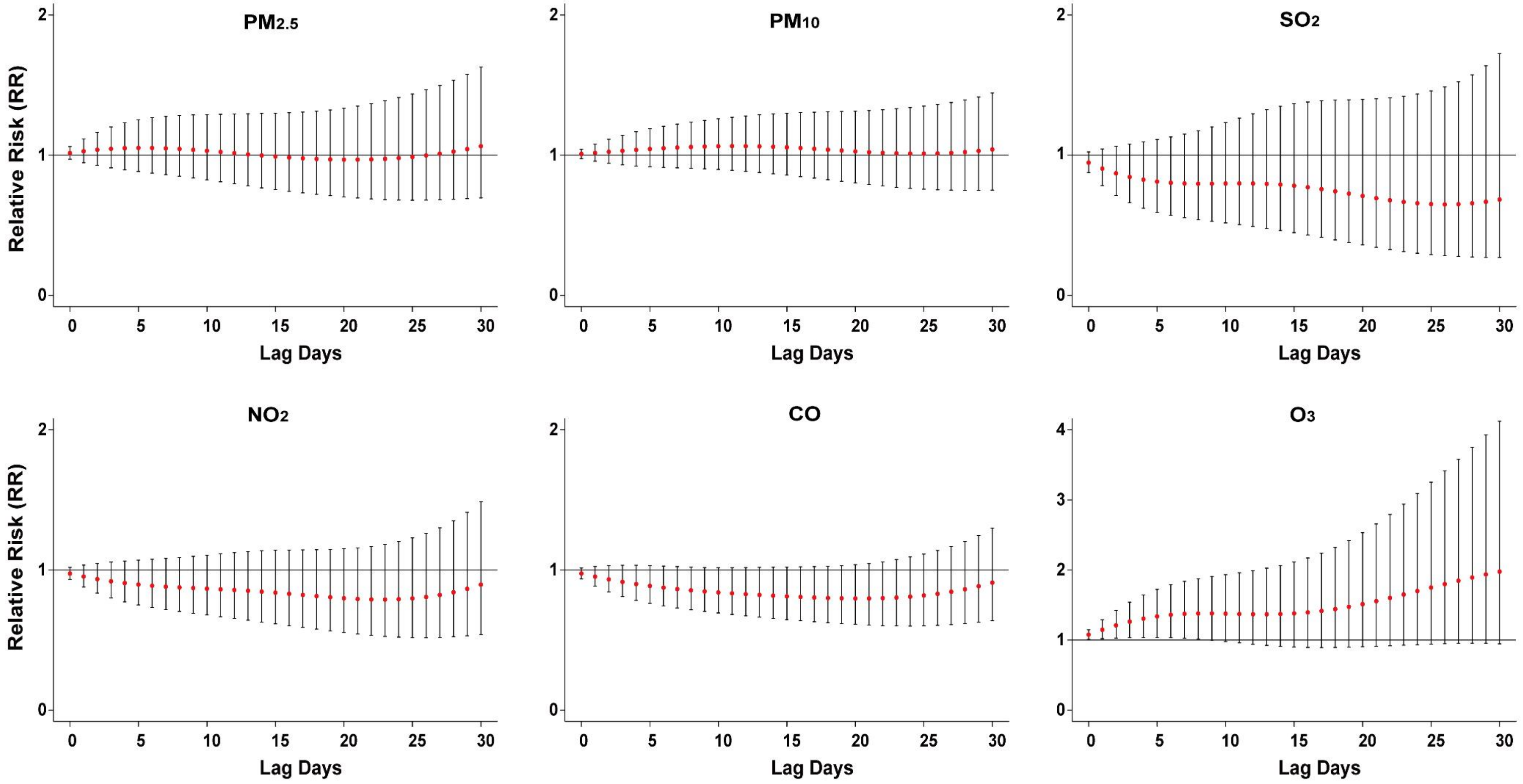

Fig. S8. The cumulative lag effect of an IQR increase in exposure to air pollutants on the RR of PTB. RR, relative risk. Lag days (day)

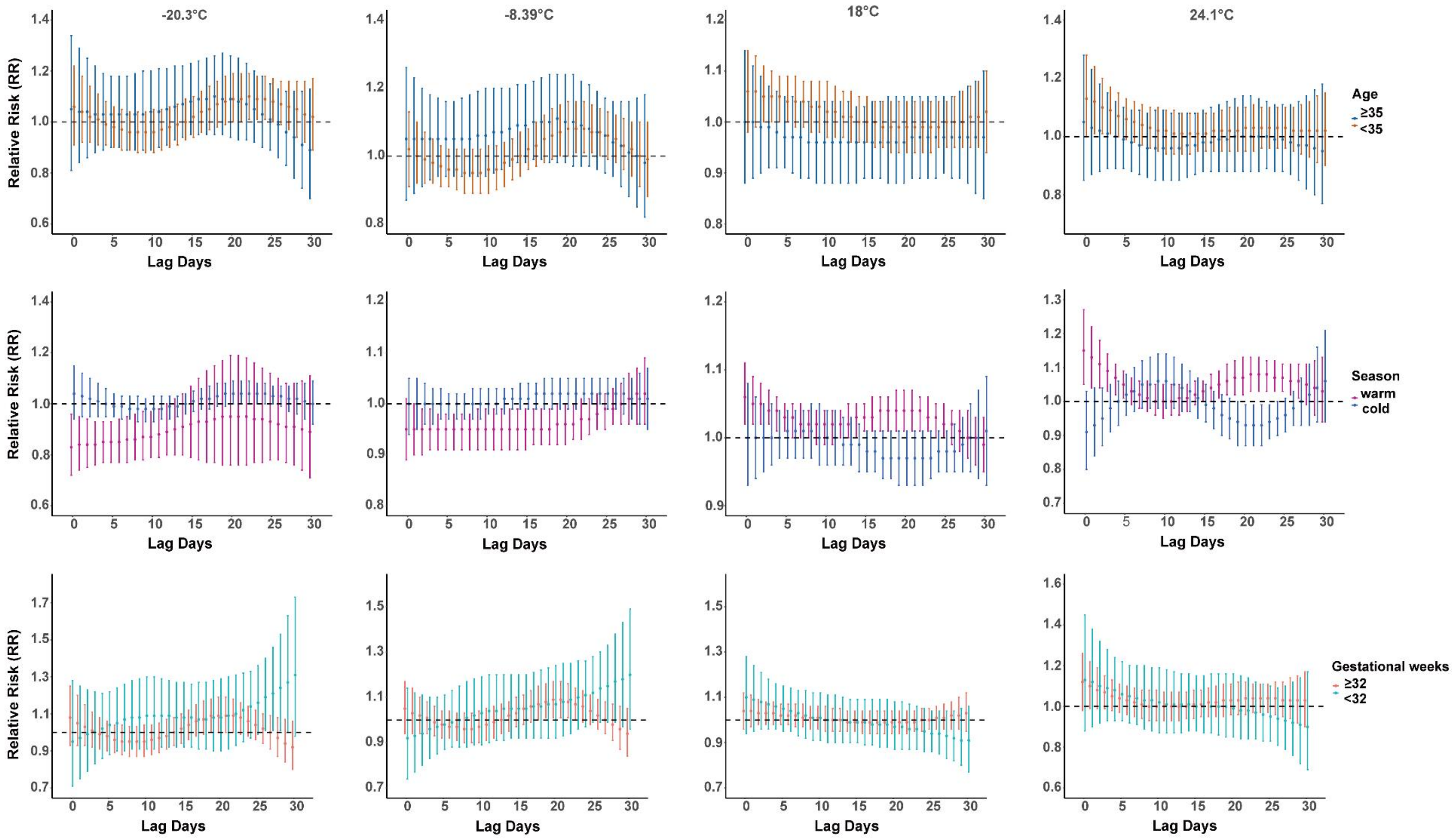

Fig. S9. Lag-specific relative risk of temperature and preterm birth (95% CI) stratified by maternal age, gestational weeks, and season. RR, relative risk. Lag days (day)

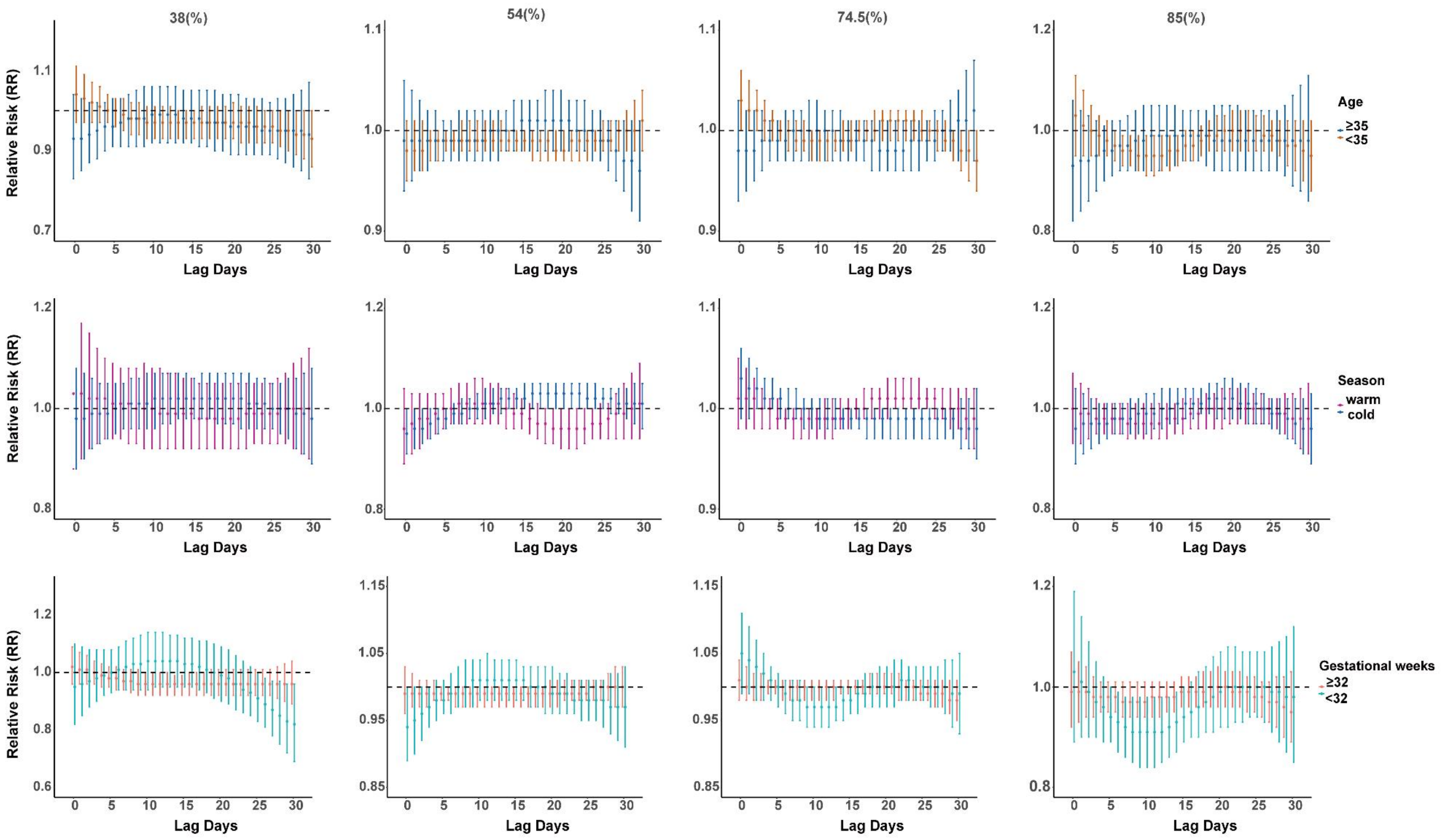

Fig. S10. Lag-specific relative risk of relative humidity and preterm birth (95% CI) stratified by maternal age, gestational weeks, and season. RR, relative risk. Lag days (day)

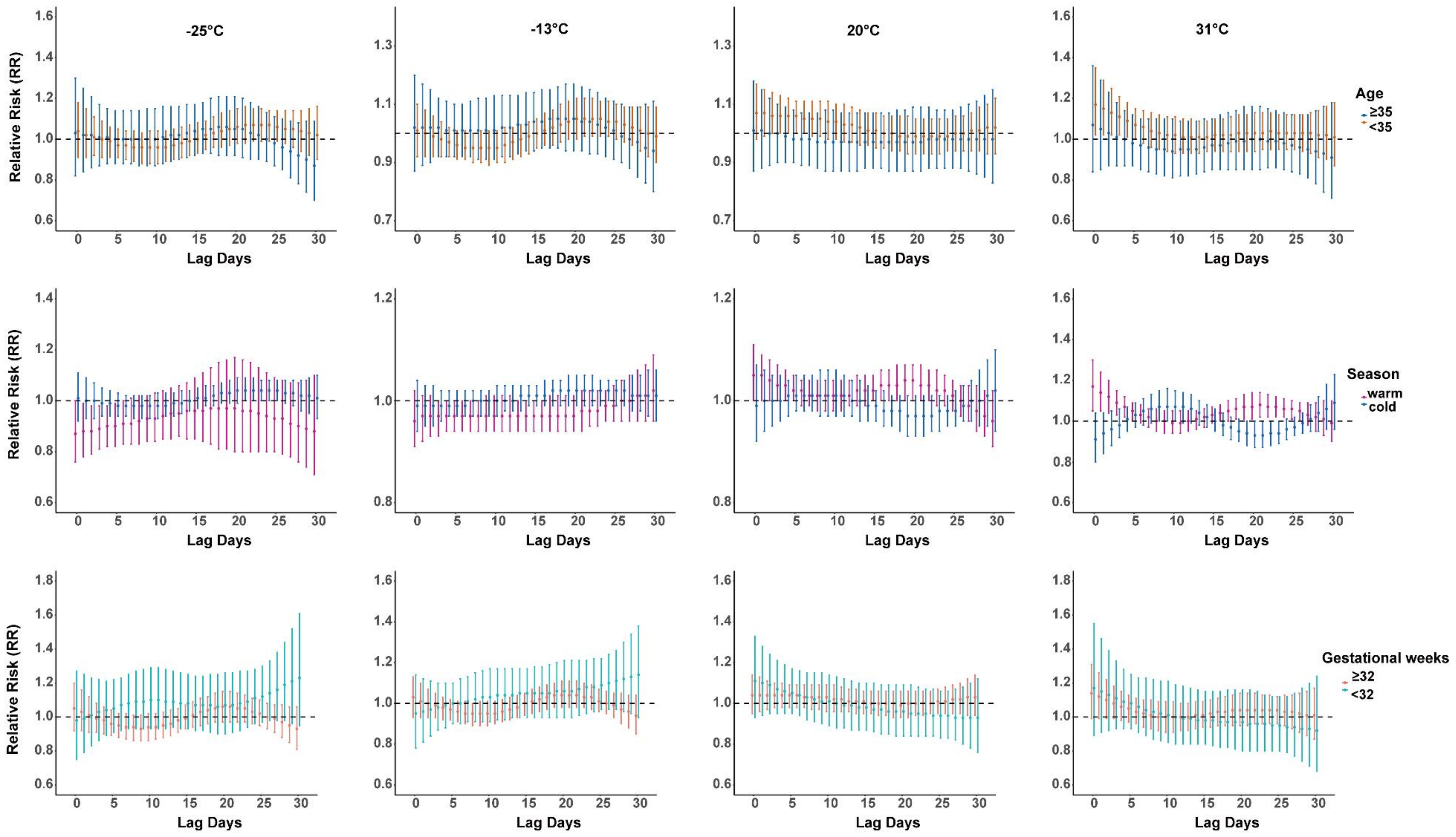

Fig. S11. Lag-specific relative risk of Humidex and preterm birth (95% CI) stratified by maternal age, gestational weeks, and season. RR, relative risk. Lag days (day)

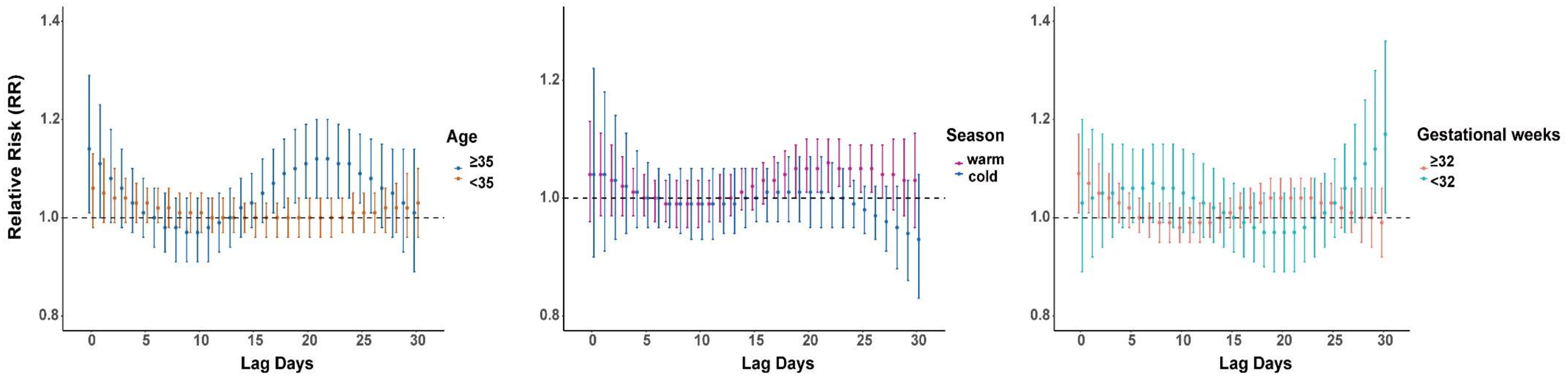

Fig. S12. Lag-specific relative risk of an IQR increase in ozone exposure and preterm birth (95% CI) stratified by maternal age, gestational weeks, and season. RR, relative risk. Lag days (day)

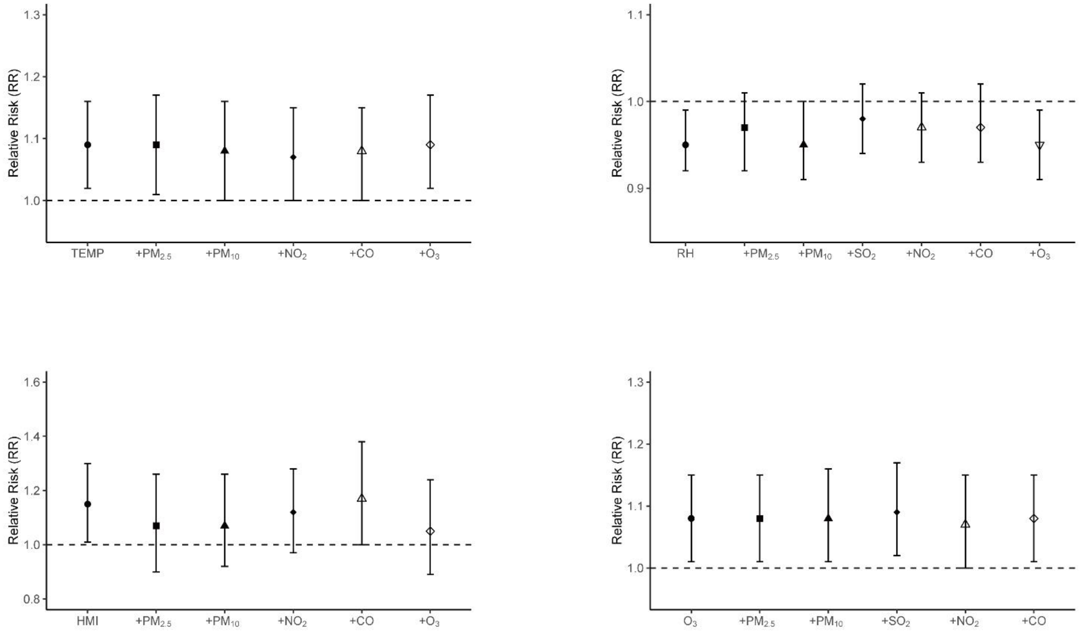

Fig. S13. The relative risk (with 95% CI) of PTB associated with temperature, relative humidity, Humidex, and ozone under multiple pollutant models

Note: The associations of PTB with low temperature (-8.39 °C) compared to the referent temperature (6.82 °C), with adjustment for air pollutants. The associations of PTB with low relative humidity (38%) compared to the referent relative humidity (64.5%), with adjustment for air pollutants. The associations of PTB with high Humidex (31 °C) compared to the referent Humidex (4 °C), with adjustment for air pollutants. The associations of PTB with an IQR increase in O_3 exposure, with adjustment for air pollutants. RR, relative risk
